# Persistent Drug Reaction With Eosinophilia and Systemic Symptoms (DRESS) Syndrome Associated With Vancomycin-Impregnated Orthopedic Cement: A Case Report

**DOI:** 10.7759/cureus.117221

**Published:** 2026-09-30

**Authors:** Trey J Dschaak

**Affiliations:** 1 Internal Medicine Residency, University of Colorado System, Aurora, USA

**Keywords:** infection, multidisciplinary, multidisciplinary decision-making, orthopedic implant related infection, total joint arthroplasty (tja), vancomycin-induced dress syndrome

## Abstract

Drug reaction with eosinophilia and systemic symptoms (DRESS) is a severe delayed hypersensitivity reaction most commonly associated with anticonvulsants and antibiotics. We describe a 50-year-old woman with chronic polymicrobial prosthetic joint infection (PJI) whose course was complicated by refractory DRESS attributed to vancomycin. Despite discontinuation of systemic vancomycin, disease activity persisted for months and required high-dose corticosteroids, mycophenolate mofetil, intravenous immunoglobulin, benralizumab, and cyclosporine. Ongoing antigen exposure from vancomycin-containing orthopedic cement and an antibiotic-coated intramedullary nail was suspected. Her prolonged hospitalization was complicated by cytomegalovirus (CMV) reactivation, steroid-induced hyperglycemia, acute kidney injury, severe edema, chronic pain, and recurrent infection. Ultimate management required removal of antibiotic cement and hardware, prolonged antimicrobial therapy, and multidisciplinary coordination. This case suggests that implanted antibiotic-containing orthopedic materials may perpetuate DRESS despite cessation of systemic drug exposure.

## Introduction

Drug reaction with eosinophilia and systemic symptoms (DRESS), also known as drug-induced hypersensitivity syndrome, is a life-threatening T-cell-mediated adverse drug reaction characterized by rash, eosinophilia, systemic symptoms, and end-organ involvement [1,2]. Vancomycin has emerged as a leading cause of DRESS among hospitalized patients [2]. Most cases improve after withdrawal of the offending agent and corticosteroid therapy. Refractory disease raises concern for persistent antigen exposure, viral reactivation, or additional medication triggers. Antibiotic-loaded orthopedic cement and antibiotic-coated implants function as local drug-delivery systems that may continue releasing antimicrobial agents after implantation [3-5]. We report a prolonged case of vancomycin-associated DRESS in a patient with prosthetic joint infection, in whom ongoing elution of vancomycin from antibiotic-loaded cement and an antibiotic-coated intramedullary nail was hypothesized to contribute to persistent disease activity despite discontinuation of systemic vancomycin [3-5].

## Case presentation

A 50-year-old woman with hypothyroidism, chronic pain, pulmonary embolism on anticoagulation, asthma, anxiety, and depression underwent right total hip arthroplasty (THA) six months prior to initial presentation. Her postoperative course was complicated by early prosthetic joint infection requiring repeated surgical interventions including prosthesis explantation, antibiotic spacer placement, resection arthroplasty, and ultimately placement of a vancomycin-containing antibiotic-coated retrograde femoral nail and polymethylmethacrylate cement in addition to IV vancomycin. 

Intravenous vancomycin was initiated for treatment of persistent prosthetic joint infection after prior therapy with piperacillin-tazobactam and daptomycin. The patient developed diffuse erythroderma, desquamation, severe pruritus, edema, purpura, and marked eosinophilia approximately one month after initiation of vancomycin. Peak absolute eosinophil count was 3.6 ×10^9^/L. Although the prior antimicrobial agents were also considered, vancomycin was favored as the most likely culprit because of its well-established association with DRESS and the temporal relationship between exposure and symptom onset. The patient met RegiSCAR criteria for probable DRESS with a total score of 4 (Table 1) [5]. Outside dermatologic evaluation led to treatment with high-dose prednisone. Vancomycin was discontinued because of concern for DRESS; however, the rash repeatedly flared whenever corticosteroids were tapered. Notably, a retained vancomycin-coated femoral nail remained in situ after discontinuation of systemic vancomycin. She was transferred to the University of Colorado Hospital six months after THA for multidisciplinary evaluation of persistent infection and refractory DRESS. 

**Table 1:** RegiSCAR Assessment for DRESS DRESS: drug reaction with eosinophilia and systemic symptoms.

RegiSCAR Criterion	Clinical Finding	Score
Fever ≥38.5°C	Documented during illness	0
Enlarged lymph nodes	Not documented	0
Atypical lymphocytes	Not documented	0
Eosinophilia	Peak absolute eosinophil count 3.6 × 10⁹/L (≥1,500 cells/μL)	+2
Skin rash involving >50% body surface area	Diffuse erythroderma involving >50% BSA	+1
At least two of edema, infiltration, purpura, or scaling	Edema, desquamation/scaling, and purpura documented	+1
Skin biopsy suggestive of DRESS	Mixed spongiotic and interface dermatitis consistent with drug eruption/DRESS	0
Internal organ involvement	No definitive DRESS-attributable organ involvement identified	0
Resolution ≥15 days	Not scored	-1
Alternative diagnoses excluded	Infectious, dermatologic, and other etiologies evaluated and excluded	+1
Total score		4 (Probable DRESS)

At transfer, she had diffuse erythroderma with desquamation, edema, purpuric lesions, severe pruritus, persistent drainage from the hip wound, leukocytosis, fevers, and debilitating pain. Histopathology from repeat skin biopsy demonstrated mixed spongiotic and interface dermatitis consistent with drug eruption/DRESS (Figure 1). In addition to the Hospital Medicine team, dermatology, orthopedic surgery, infectious diseases, allergy/immunology, endocrinology, and addiction medicine were involved in management. Initial therapy included prednisone up to 100 mg daily and mycophenolate mofetil, but symptom control remained poor. 

**Figure 1:**
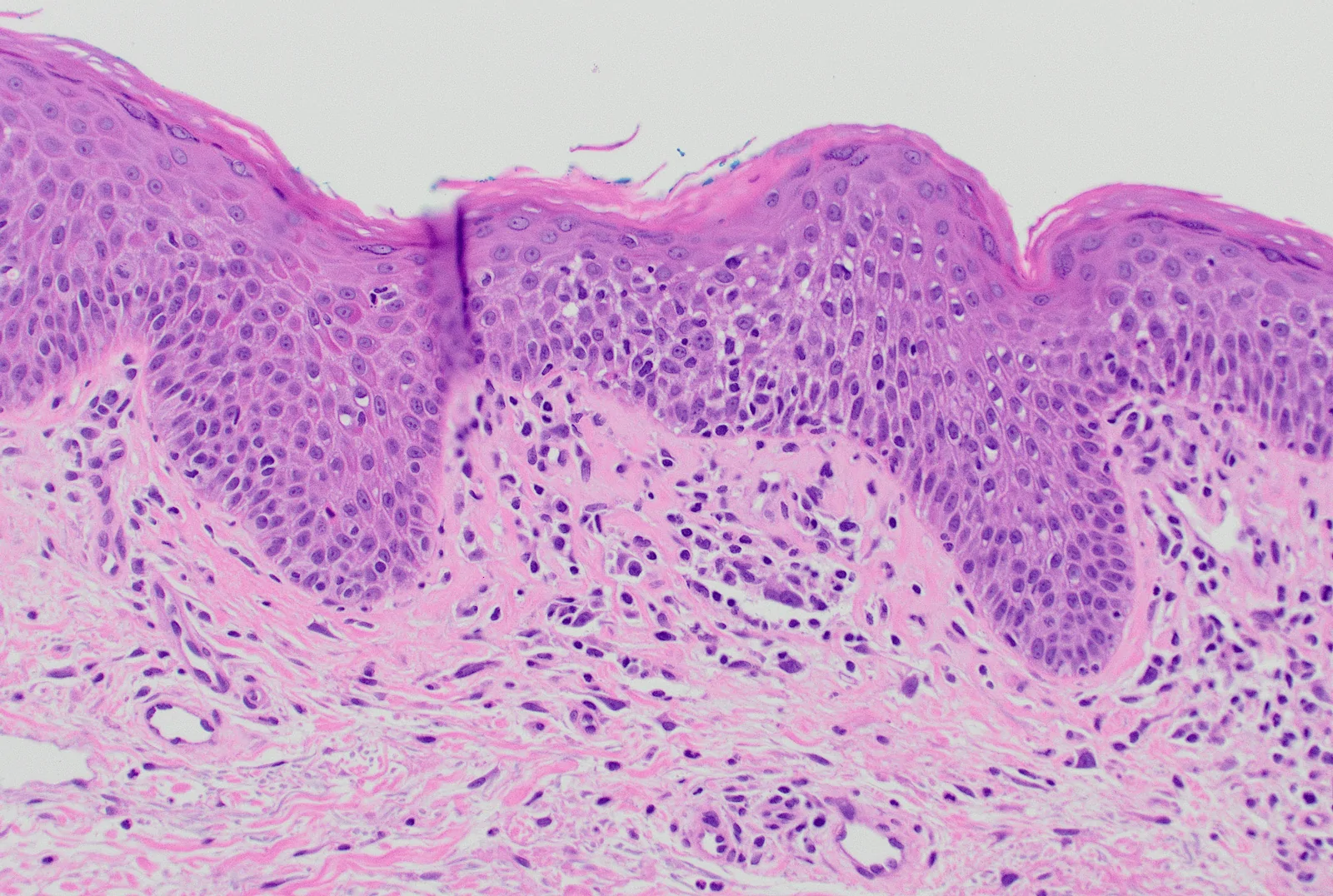
Hematoxylin and eosin stained image at 100x demonstrated a mixed inflammatory reaction with spongiosis and interface dermatitis. The presence of multiple inflammatory reaction patterns strongly supports a cutaneous drug eruption and is compatible with the clinical diagnosis of DRESS. DRESS: drug reaction with eosinophilia and systemic symptoms.

Microbiologic data demonstrated an extraordinarily complex polymicrobial infection in the right hip joint. Organisms isolated throughout her course included *Pseudomonas aeruginosa*, *Escherichia coli*, *Enterococcus faecalis*, vancomycin-resistant *Enterococcus faecium*, *Corynebacterium striatum*, *Bacteroides fragilis*, and *Candida glabrata*. Despite prolonged antimicrobial therapy, she continued to experience wound drainage, recurrent fevers, elevated inflammatory markers, and positive cultures.

The prolonged disease course was marked by multiple complications. CMV viremia developed with PCR levels exceeding 17,000 IU/mL, necessitating valganciclovir therapy. Steroid-induced hyperglycemia required insulin treatment. The patient experienced acute kidney injury, severe anasarca, chronic wound drainage, mobility limitations, chronic pain requiring escalation of opioid therapy and eventual buprenorphine induction, as well as profound psychosocial distress from her prolonged hospitalization. Acute kidney injury was considered multifactorial and was not definitively attributed to DRESS-related organ involvement.

Given continuation of symptoms despite discontinuation of systemic vancomycin, concern arose regarding persistent exposure from vancomycin-laden orthopedic materials. As summarized in Table 2, disease activity persisted despite discontinuation of systemic vancomycin, worsened following implantation of an antibiotic-laden cement-coated nail, and demonstrated initial improvement following removal of retained orthopedic materials. Additional therapies included four days of intravenous immunoglobulin, two doses of benralizumab, and escalation to cyclosporine.

After extensive multidisciplinary discussion, the patient underwent right hip irrigation and debridement, removal of the retrograde intramedullary nail, removal of antibiotic cement, partial proximal femur resection, and wound-vac placement. Repeat debridement and closure followed two days later. Operative cultures grew *Corynebacterium striatum* and *Enterococcus faecium*. Initial dermatologic improvement was observed within several days of hardware and cement removal. However, disease activity subsequently flared when prednisone was tapered to 40 mg daily, raising concern for additional medication triggers, including trimethoprim-sulfamethoxazole and meropenem. Because no recurrent *Pseudomonas *or other aerobic gram-negative organisms had been isolated, empiric coverage with meropenem was discontinued. Daptomycin, meropenem, and trimethoprim-sulfamethoxazole were subsequently stopped, and antimicrobial therapy was adjusted to tedizolid, metronidazole, micafungin, atovaquone, and valganciclovir. Ultimately, the rash improved on cyclosporine with gradual corticosteroid tapering. A chronology of major antimicrobial exposures, orthopedic procedures, DRESS-related events, and therapeutic interventions is summarized in Table 2.

**Table 2:** Timeline of Clinical Events, Antimicrobial Exposures, DRESS Disease Activity, and Therapeutic Interventions DRESS: drug reaction with eosinophilia and systemic symptoms.

Time Relative to Vancomycin Initiation	Clinical Event
Day -61	First-stage revision performed. Postoperatively treated with piperacillin-tazobactam and daptomycin.
Day 1	Intravenous vancomycin initiated for treatment of persistent prosthetic joint infection, with a planned 6-week treatment course.
Prior to Day 34	Development of diffuse erythroderma, desquamation, severe pruritus, edema, purpura, and eosinophilia that culminated in hospitalization for evaluation of DRESS.
Day 34	Hospitalized for rash concerning for DRESS; vancomycin discontinued and antimicrobial therapy changed to linezolid.
Day 37	Resection of right hip arthroplasty with irrigation and debridement.
Day 50	Debridement of the right femoral shaft with retrograde insertion of an antibiotic-laden cement-coated femoral nail for fracture fixation. Subsequent worsening of rash was noted; symptoms improved with prednisone 100 mg daily.
Day 80	Joint aspiration cultures positive for vancomycin-resistant *Enterococcus faecium *and *Candida glabrata*.
Day 141	Transfer to the University of Colorado Hospital. Skin biopsy demonstrated mixed spongiotic and interface dermatitis consistent with drug eruption/DRESS.
Day 145	Intravenous immunoglobulin administered.
Days 145 and 171	Benralizumab administered.
Day 185	Cyclosporine initiated.
Day 220	Removal of antibiotic cement and intramedullary nail.
Day 224	Initial dermatologic improvement observed following hardware and cement removal.
Day 226	Disease flare occurred during prednisone tapering to 40 mg daily, raising concern for additional medication triggers, including trimethoprim-sulfamethoxazole and meropenem.
Day 228	Daptomycin, meropenem, and trimethoprim-sulfamethoxazole discontinued. Antimicrobial therapy transitioned to tedizolid, metronidazole, micafungin, and atovaquone.
Subsequent course	Rash ultimately improved on cyclosporine with gradual corticosteroid tapering.

## Discussion

Persistent or relapsing DRESS after withdrawal of the inciting medications has been described and may result from prolonged immune dysregulation, viral reactivation, corticosteroid tapering, or exposure to additional medications. Therefore, continued symptoms do not by themselves establish ongoing antigen exposure. In the present case, however, the presence of vancomycin-containing cement and an antibiotic-coated nail provided a biologically plausible explanation for continued immune activation [2-4]. As summarized in Table 2, the patient's rash developed during intravenous vancomycin therapy, persisted despite discontinuation of systemic vancomycin and continued presence of vancomycin-containing orthopedic materials, worsened following implantation of an antibiotic-laden cement-coated nail, and demonstrated partial improvement after removal of retained orthopedic materials. Although these observations do not establish causality, the temporal relationship supported consideration of retained antibiotic-containing materials as one potential contributor to the patient's prolonged disease course.

The case also demonstrates the difficulty of balancing immunosuppression against active infection. The patient required escalating immunomodulatory therapy to control DRESS while simultaneously harboring a chronic polymicrobial prosthetic joint infection requiring prolonged antimicrobial therapy and repeated surgical intervention. Clinicians faced competing priorities of suppressing immune-mediated disease while preserving host defenses necessary for infection control and wound healing.

Another important feature of this case was the development of cytomegalovirus (CMV) reactivation during treatment. CMV reactivation has been associated with disease flares and increased severity in DRESS and may contribute to a prolonged or relapsing clinical course [6]. In addition to CMV viremia, the patient experienced several complications related to both severe DRESS and its treatment, including steroid-induced hyperglycemia, acute kidney injury, severe edema, chronic pain, impaired mobility, and significant psychosocial distress. Notably, the patient's acute kidney injury was considered multifactorial and was not definitively attributed to DRESS-related organ involvement. Recognition of these complications is important because morbidity in DRESS frequently extends beyond its cutaneous manifestations.

Finally, this case highlights the challenges of managing refractory DRESS and the evolving role of adjunctive therapies. Despite treatment with high-dose corticosteroids, mycophenolate mofetil, intravenous immunoglobulin, and benralizumab, meaningful and sustained improvement occurred only after a combination of cyclosporine therapy and operative source control. Emerging evidence supports the use of IL-5-targeted therapies such as benralizumab in selected cases of refractory DRESS; however, this intervention did not result in substantial clinical improvement in our patient [7,8].

Whether hardware removal directly altered disease activity cannot be definitively proven. Although the temporal relationship between removal of antibiotic-containing materials and partial clinical improvement is noteworthy, alternative explanations, including CMV reactivation, corticosteroid taper-associated relapse, ongoing immune dysregulation, and additional medication exposures, must also be considered.

## Conclusions

This case illustrates the challenges of managing severe, refractory DRESS in the setting of chronic prosthetic joint infection. Clinicians should evaluate persistent antigen exposure, viral reactivation, corticosteroid taper-associated relapse, and alternative medication triggers when DRESS remains active despite withdrawal of the suspected offending agent. Retained antibiotic-containing orthopedic materials may represent a potential contributing factor in selected patients, although causality cannot be established from this case alone. Further reports are needed to clarify the role of implanted antibiotic-delivery systems in persistent or relapsing DRESS.
